# Ozonated Sunflower Oil (OSO) Alleviates Inflammatory Responses in Oxazolone-Induced Atopic Dermatitis (AD)-Like Mice and LPS-Treated RAW 264.7 Cells

**DOI:** 10.4014/jmb.2310.10037

**Published:** 2024-01-12

**Authors:** Su-Young Kim, Jung Ok Lee, Sue Lee, Jihye Heo, Kyung-Hyun Cho, Ashutosh Bahuguna, Kwang-Ho Yoo, Beom Joon Kim

**Affiliations:** 1 Department of Dermatology, College of Medicine, Chung-Ang University, Seoul 06974, Republic of Korea; 2 Department of Medicine, Graduate School, Chung-Ang University, Seoul 06973, Republic of Korea; 3 Raydel Research Institute, Medical Innovation Complex, Daegu 41061, Republic of Korea

**Keywords:** Ozonated sunflower oil (OSO), Atopic dermatitis (AD), filaggrin, TSLP, inflammation, RAW 264.7

## Abstract

Ozone, a highly reactive oxidant molecule, is widely used as a complementary therapy for various skin diseases, including wound healing, pressure ulcers, diabetic foot, and infections. However, there is limited research on the effectiveness of ozone for atopic dermatitis (AD). Ozonated sunflower oil (OSO) is an active ingredient obtained from partially ozonated sunflower oil (SO). OSO markedly reduced the LPS-induced increase in IL-1β and nitric oxide (NO) levels in RAW 264.7 mouse macrophage cells. Oxazolone (OXZ) was applied to hairless mice to induce AD-like skin symptoms and immune response. OSO significantly alleviated the OXZ-induced increases in the number of infiltrating mast cells, epidermal thickness, AD symptoms, thymic stromal lymphopoietin (TSLP), and filaggrin, as well as the serum levels of NO, IgE, IL-1β, and TNF-α. Furthermore, OSO inhibited the IL-4/STAT3/MAPK pathway and the expression of NF-κB. Our results suggest that OSO treatment could relieve AD-mediated skin damage through its anti-inflammatory and antioxidant activities. Therefore, it can be used as a therapeutic agent against AD-related skin diseases.

## Introduction

Atopic dermatitis (AD) is a chronic inflammatory skin disease [1, 2]. In Europe and the United States, approximately 20% of children and 7–14% of adults suffer from AD [3], and the prevalence is continuously increasing. Various symptoms, including skin dryness, eczema, pruritus, and cracking, severely interfere with the quality of life of AD patients [4, 5]. An imbalance between CD4^+^ T helper type 1 (Th1) and CD4^+^ T helper type 2 (Th2) cells mainly mediates the inflammation in AD [6, 7]. Specifically, skin exposure to exogenous antigens causes the overexpression of Th2-related cytokines, including interleukin-4 (IL-4), IL-5, and IL-13, which subsequently induce B cells to produce immunoglobulin E (IgE). Increased IgE mediates histamine exocytosis in skin mast cells, resulting in edema and itching, which further aggravate AD [6, 8].

Corticosteroids, calcineurin inhibitors (*e.g.*, cyclosporine), anti-histamines, and Janus kinase inhibitors (*e.g.*, upadacitinib and abrocitinib) are major treatments for AD [9, 10]. Although these medications may relieve certain symptoms, prolonged steroid use may lead to skin thinning with subsequent bleeding, hypertension, and liver and kidney toxicity [11]. Accordingly, there is a growing need for new biological drugs that are both effective and safe.

The epidermis, the outermost layer of the skin, provides a functional barrier to the body. Epidermal damage has been considered an initial step in the development of AD [12]. Filaggrin is an essential structural protein in the stratum corneum (SC), which is the main component of the epidermal skin barrier. It can enhance the terminal differentiation of the epidermis and the formation of the skin barrier [13, 14]. Downregulation of filaggrin in the skin or mutations with loss of function in the filaggrin gene have been observed in AD patients [15]. Furthermore, it is known that the increased thymic stromal lymphopoietin (TSLP) in the epidermis of AD patients activates extracellular-signal-regulated kinase [ERK, belonging to the mitogen-activated protein kinase (MAPK) family] and signal transducer and activator of transcription 3 (STAT3), leading to the inhibition of filaggrin expression [16]. Therefore, for multifaceted reasons, the integrity of the epidermal barrier is compromised in AD.

Ozone (O_3_), a highly reactive oxidant molecule, affects various processes, including immunomodulation, inflammation, antioxidant activity, antibacterial responses, and epigenetic modifications, as well as analgesic, biosynthetic, and vasodilative functions. It also acts as a modulator of regenerative pathways [17-19]. Based on these properties, ozone has been used as a complementary therapy for several cutaneous diseases such as acne, wound healing, psoriasis, diabetic foot, and pressure ulcers [17]. In addition, ozone therapy has the advantage of being a non-invasive and cost-effective treatment [17]. J. Zeng *et al*. have reported that topical ozone therapy in AD patients alters the proportional ratio of *Staphylococcus* and *Acinetobacter*, restoring microbial diversity in AD lesions and decreasing SCORAD scores [20]. It is well known that sunflower oil (SO) itself moisturizes and protects damaged skin barriers. Especially, ozonated sunflower oil (OSO) is a mixture of active ingredients obtained from the partial ozonation of SO. According to a previous study, OSO has an anti-inflammatory effect on the skin diseases of mice and humans [21, 22]. However, the effectiveness of OSO on AD has not been thoroughly investigated. Therefore, in this study, we investigated the potential therapeutic effects of OSO using AD-like mouse models and LPS-treated RAW 264.7 murine macrophage cells, which are commonly used to observe inflammatory responses. Also, identified the mechanism responsible for these effects.

## Materials and Methods

### Ozonated Sunflower Oil (OSO)

OSO (Raydel Bodyone Flambo oil) was obtained from Rainbow and Nature Pty, Ltd. (Australia). Its physicochemical characteristics were in the typical range of Oleozon, as described elsewhere [23, 24]: 783.4 mmol of peroxide/kg (range, 500–800 mmol/kg) and a viscosity of 131.5 mPa.s (90–350 mPa.s), with an optimal acidity of 2.42 mg KOH/g. Commercially available SO (Ondoliva oil, Spain) was purchased from a local market.

### 1,1-Diphenyl-2-Picrylhydrazyl (DPPH) Assay

OSO, SO, or Ascorbic acid (AA, Sigma, USA) were dissolved in dimethyl sulfoxide (DMSO) and mixed with a DPPH solution (120 μM). AA was used as a positive control, while SO was used as the control. The reaction mixture was kept in dark for 3 h at room temperature (RT), and the change in absorbance was measured at 517 nm. The inhibition ratio (%) was calculated as 100 – [(OD of sample / OD of control) × 100].

### Murine Macrophage RAW 264.7 Cell Culture and Reagents

Murine macrophage RAW 264.7 cells were obtained from the Korean Cell Line Bank (KCLB No. 40071) and cultured in Dulbeccós Modified Eaglés Medium (DMEM) (Welgene, Republic of Korea) supplemented with 1%(v/v) penicillin-streptomycin and 10% (v/v) fetal bovine serum (FBS; Gibco) at 37°C with a 5% CO_2_ atmosphere. Initially, OSO and SO were diluted to a 50% concentration using DMSO (Sigma), and then serially diluted to 0.01% using DMEM.

### Cell Viability Assay

RAW 264.7 cells were treated with OSO for 24 h, and cell viability was assessed using a WST-8 assay kit (Biomax, Republic of Korea).

### Measurement of IgE, IL-1β, TNF-α, and Nitric Oxide (NO) Concentrations

Serum samples were collected from whole blood using a Mini Collect tube (Greiner bio-one, Austria). IgE, IL-1β, and TNF-α levels in the serum were measured using an enzyme-linked immunosorbent assay (ELISA) kit (Invitrogen, USA). RAW 264.7 cells were cultured with OSO or lipopolysaccharide (LPS, 1 μg/ml, Sigma) and incubated for 24 h. The cell culture medium was centrifuged and the supernatants were collected. IL-1β and NO levels were analyzed using an ELISA kit (Thermo Fisher Scientific) and a Nitrate Plus Detection kit (iNtRON Biotechnology, Republic of Korea) following the manufacturer’s instructions.

### Induction of AD-Like Lesions (Sensitization and Challenge)

The induction of AD in hairless mice using OXZ (oxazolone) was performed following the method described by Man *et al*. [25]. To sensitize the mice, 10 μl of 5% OXZ (dissolved in EtOH) was applied to the skin on the back of their necks 2 weeks before the start of the experiment (Sensitization step). After a week, 60 μl of 0.3% OXZ was applied to the lower backs of the mice, three times per week for 3 weeks (Challenge step), followed by daily topical administration of OSO for 2 weeks.

### Animals and Animal Experimental Design (Groups 1-6)

Hairless mice (6-week-old females) were purchased from Saeron Bio Inc. (Republic of Korea) and acclimated for 1 week under the following conditions: 23 ± 2°C, 55 ± 10% humidity, and 12-h-light/12-h-dark cycle. This study protocol was reviewed and approved by the Chung-Ang University Animal Laboratory Ethics Committee, approval number 202100029. The normal group (Group 1) received no treatment. The OXZ (Sigma)-only group (Group 2) received OXZ alone. The OXZ + DEX group (Group 3) received OXZ and dexamethasone (DEX, Sigma) (0.03% in ethanol, EtOH). The OXZ + Vehicle group (Group 4) received OXZ and 100% SO. The other groups included the OXZ + 10% OSO (Group 5) and the OXZ + 100% OSO (Group 6). DEX, Vehicle, and OSO were topically applied 2 h after the application of OXZ. After 2 weeks, we administered the drugs to all of the groups (*n* = 4 per group) daily for 2 weeks at a dosage of 100 μl on the AD lesion.

### Measurement of Dermatitis Severity

The anesthetic consisting of Zoletil (0.008 cc/10 g; 40 mg/kg) and Rompun (0.002 cc/10 g; 5 mg/kg) was diluted (10-fold) with normal saline. The dorsal skin of the anesthetized test animals was photographed using a digital single-lens reflex camera (Nikon, Japan) in close proximity. At the end of the experimental period, the severity of AD-like dorsal skin lesions was assessed using the dermatitis score as described by Kang *et al*. [25]. Each symptom, including excoriation, scaling, edema, and erythema, was scored on a scale of 0 (almost clear), 1 (mild), 2 (moderate), and 3 (severe). The clinical skin score, which is defined as the sum of the individual scores, ranged from 0 to 12. Dermatitis severity was evaluated by two independent observers.

### Body Weight, Spleen Weight, and Lymph Node Size

The mice were weighed weekly during the experiment. At the end of the experimental period, the weight of the spleen and the size of the inguinal lymph nodes were measured. The spleen weight was normalized to the body weight of each mouse.

### Histological Analysis

Skin biopsies were fixed in 10% formalin for 24 h. Paraffin-embedded 5-μm-thick sections were cut, mounted on POLYSINE Slides (Thermo Fisher Scientific), dewaxed in xylene, and then dehydrated using an ethanol series. Hematoxylin and eosin (H&E) staining was performed to examine histological features and skin thickness. Toluidine blue (TB) staining was performed to visualize skin mast cells. Additionally, tissue slides were stained with primary antibodies listed in Table 1. The slides were washed with phosphate buffered saline with Tween 20 (PBS-T) and incubated with a two-component high-sensitivity 3,3-diaminobenzidine (DAB) chromogenic substrate (Vector Laboratories USA). After washing, the slides were dehydrated and mounted using a permount mounting medium (Thermo Fisher Scientific). All stained tissue slides were photographed using a slide scanner (Pannoramic MIDI; 3DHISTECH Ltd, Hungary) and analyzed using the Case Viewer software.

### Transepidermal Water Loss (TEWL) and Corneometer

At the end of the experiment, the TEWL (g/m^2^h) and hydration levels (arbitrary units, A.U.) in the stratum corneum (SC) were measured using a Tewameter (Courage Khazaka Electronic GmbH, Germany) and a Corneometer CM 825 (Courage Khazaka Electronic GmbH), respectively. The measurements were performed at RT and a humidity level of 50–60%. Each measurement was recorded three times (excluding the initial value), and the average value was documented.

### Western Blot Analysis

The skin proteins were extracted using the PRO-PREP Protein Extraction Solution (iNtRON Biotechnology). Equal amounts of total protein were electrophoresed on 10% SDS PAGE gels and transferred to nitrocellulose membranes (Cytiva, USA). The membranes were blocked with 5% fat-free milk in Tris-buffered saline with 0.1%Tween 20 Detergent (TBS-T) at RT for 1 h. Subsequently, the membranes were incubated overnight at 4°C with primary rabbit antibodies listed in Table 1. After incubation with secondary antibodies (Vector Laboratories Inc., USA), immunodetection was performed using an EzWestLumi plus kit (ATTO Corporation, Japan) according to the manufacturer's protocol. The protein bands were visualized using EZ-capture II (ATTO Corporation) and Image Saver 5.0 program (ATTO corporation) according to the manufacturer’s instructions and analyzed using the Image J software (USA).

### Statistical Analysis

Data are presented as the mean ± standard error of the mean (SEM) from at least three independent experiments. Data analyses were performed using unpaired one-way analysis of variance (ANOVA) followed by variance and the Tukey post-hoc test. Statistical analysis was performed using GraphPad Prism 7.0 software (GraphPad Software Inc., USA). Differences with p values lower than 0.05 were considered statistically significant and indicated with the following symbols: *, *p* < 0.05; **, *p* < 0.01; and ***, *p* < 0.001.

## Results

### Antioxidant (in vitro) and Anti-Inflammatory Effects of OSO in RAW 264.7 Cells

**Fig. 1:**
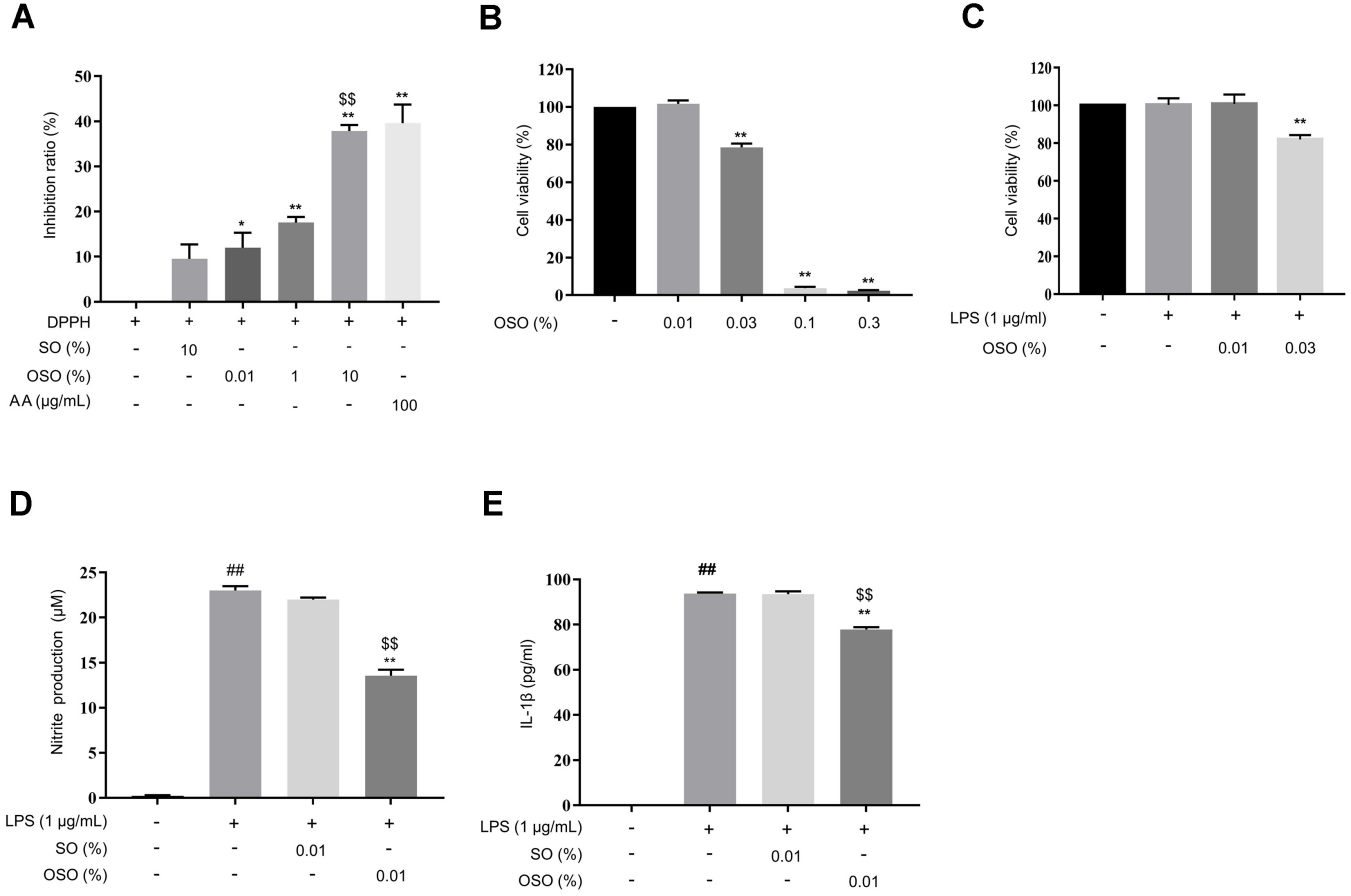
Anti-inflammatory and antioxidant effects of OSO on LPS-stimulated RAW 264.7 cells. (**A**) DPPH radical scavenging activity of OSO at various concentrations. (**B-C**) Viability of RAW 264.7 cells was measured using a WST-8 assay. The cells were treated with OSO for 24 h (**B**) or co-treated with OSO and LPS for 24 h (**C**). (**D-E**) NO assay and ELISA were performed to determine NO (**D**) and IL-1β (**E**) levels in the culture supernatant after treatment with OSO in the presence of LPS for 24 h. The results are expressed as the mean ± SEM (*n* = 3). *, *p* < 0.05; **, *p* < 0.01 compared with DPPH-only. ^$$^, *p* < 0.01 compared with DPPH+SO (**A**). **, *p* < 0.01 compared with control (**B**). **, *p* < 0.01 compared with LPS-only (**C**). **, *p* < 0.01 compared with LPS-only. ^$$^, *p* < 0.01 compared with LPS + SO. ^##^, *p* < 0.01 compared with the non-treated samples (**D–E**).

The DPPH radical scavenging activity increased upon OSO treatment in a dose-dependent manner. Exposure to 10% OSO inhibited 37% of the DPPH radical, which was similar to the 39% inhibition observed with AA (100 μg/ml) (Fig. 1A). As shown in Fig. 1B, the viability of RAW 264.7 cells was not significantly affected by 0.01%OSO. To confirm the anti-inflammatory effect of OSO in RAW 264.7 cells, 0.01% OSO and LPS (1 μg/ml), which showed no cytotoxicity, were selected (Fig. 1C). Excessive production of NO indicates an inflammatory response [26]. OSO significantly inhibited the LPS-mediated overproduction of NO. Levels of NO decreased by 43% upon treatment with 0.01% OSO compared to the non-treated control group (Fig. 1D). IL-1β, a pro-inflammatory cytokine, mediates acute inflammation [27]. IL-1β levels were significantly lower in cells co-treated with LPS and OSO compared to those treated with LPS only (Fig. 1E). These observations indicate that 0.01% OSO has antioxidant and anti-inflammatory activities against LPS-induced inflammation in RAW 264.7 cells.

### OSO Improves AD in the OXZ-Induced AD-Like Hairless Mouse Model

**Fig. 2:**
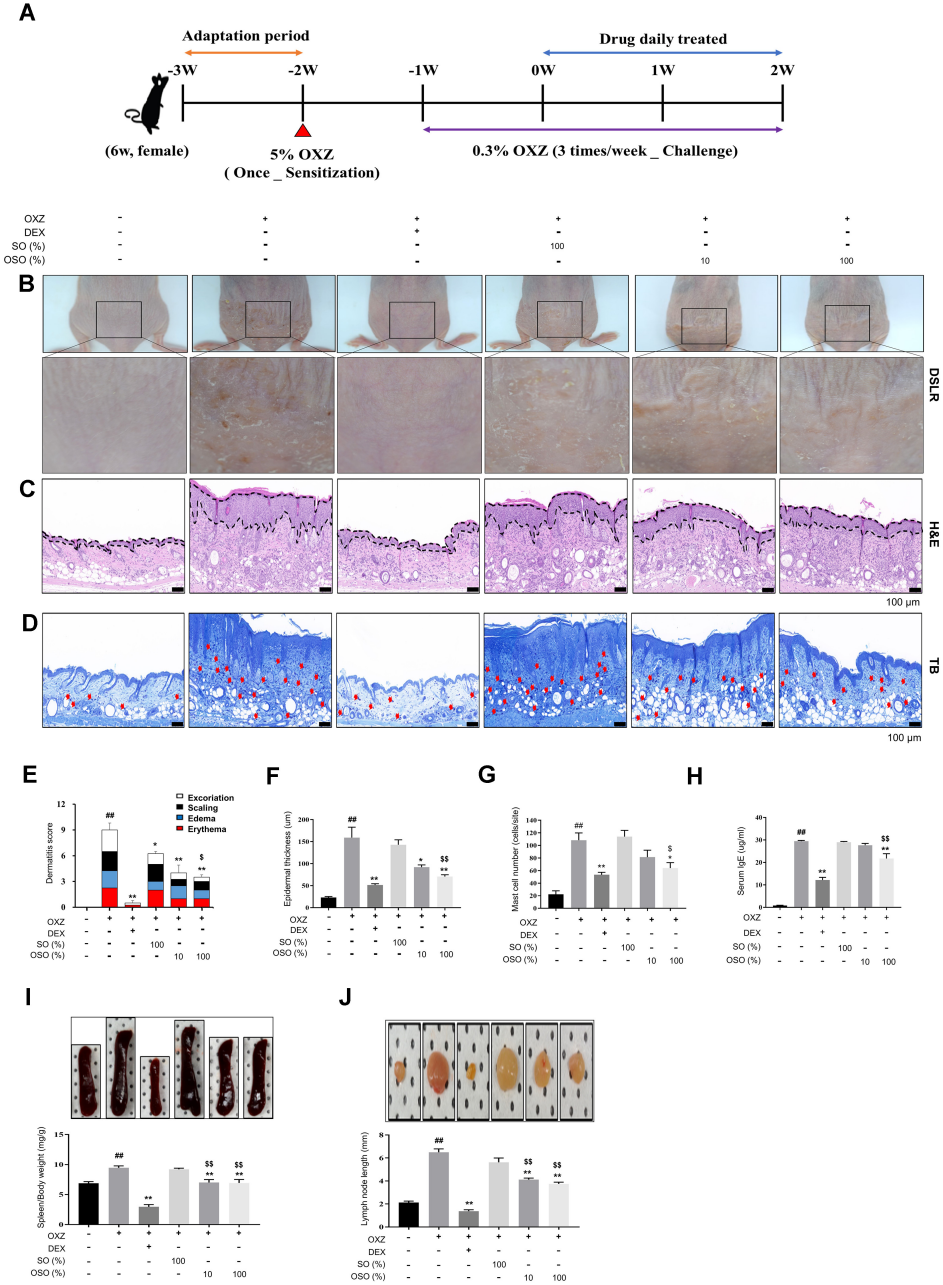
Topical administration of OSO improves AD symptoms. (**A**) Experimental procedure. (**B**) Representative photographs of mouse dorsal skin. (**C**) Histological examination of dorsal skin lesion epidermal thickness using H&E staining. Scale bar, 100 μm. (**D**) Number of infiltrated mast cells as determined using TB staining. Scale bar, 100 μm. (**E**) Dermatitis score was analyzed by summing the observed signs in the clinical trials, such as excoriation, scaling, edema, and erythema, with scores of 0 (almost clear), 1 (mild), 2 (moderate), and 3 (severe) for each sign. (**F**) Histological examination of dorsal skin lesion epidermal thickness was measured using H&E staining. Scale bar, 100 μm. (**G**) Number of infiltrated mast cells was measured using TB staining. Scale bar, 100 μm. (**H**) ELISA was performed to determine IgE levels in the serum. (**I–J**) At the end of the experiment, the mice were euthanized, and spleen weight (**I**) and inguinal lymph node length (**J**) were measured. The results are expressed as the mean ± SEM (*n* = 3–4 per group). *, *p* < 0.05; **, *p* < 0.01 compared with the OXZ-only group. $, *p* < 0.05; ^$$^, *p* < 0.01 compared with the OXZ + SO group. ^##^, *p* < 0.01 compared with the normal group (**E-J**).

To investigate the effectiveness of OSO in improving AD symptoms, mice were sensitized with 5% OXZ on the back of their neck skin -3 weeks prior to challenges on the lower part of their back with 0.3% OXO for 3 weeks (Fig. 2A). The clinical characteristics of AD, including excoriation, scaling, edema, and erythema, were evaluated and scored. The dermatitis score significantly increased to 9.00 ± 0.82 in the OXZ-only group compared to the normal group (*p* < 0.01). However, it decreased significantly to 4.00 ± 0.91 (*p* < 0.01) and 3.50 ± 0.29 (*p* < 0.01) in the OXZ + 10% OSO and OXZ + 100% OSO groups, respectively (Fig. 2B and 2E). Histologic evaluation of the skin tissue using H&E staining revealed that the epidermal thickness of the OSO-administered group significantly decreased to 42.23% (*p* < 0.01) and 55.62% (*p* < 0.01), respectively, in the OXZ + 10% OSO and OXZ + 100% OSO groups compared with the OXZ-only group (Fig. 2C and 2F). The number of infiltrated mast cells in the dermis layer significantly decreased to 64.0 ± 14.93 cells/site in the OXZ + 100% OSO group compared to the OXZ-only group (*p* < 0.05; Fig. 2D and 2G). Mice in the OXZ-sensitized group had elevated IgE levels in their serum. In contrast, the mice in the OXZ + 100% OSO group exhibited reduced IgE levels, indicating relief from inflammation (*p* < 0.01; Fig. 2H). Furthermore, to evaluate whether OSO decreases systemic immune response, the size of the inguinal lymph nodes and spleen weight were measured. Compared with the OXZ-only group, spleen weights in the OXZ + 10% OSO and OXZ + 100% OSO groups were significantly decreased by 26% (*p* < 0.01) and 26.76% (*p* < 0.01), respectively (Fig. 2I). Inguinal lymph node size was significantly decreased to 4.1 ± 0.25 mm in the OXZ+ 10% OSO (*p* < 0.01) and to 3.8 ± 0.29 mm in the OXZ + 100% OSO groups (*p* < 0.01) compared to the OXZ-only group (Fig. 2J).

### OSO Restores Skin Barrier Function in an AD-Like Hairless Mouse Model

**Fig. 3:**
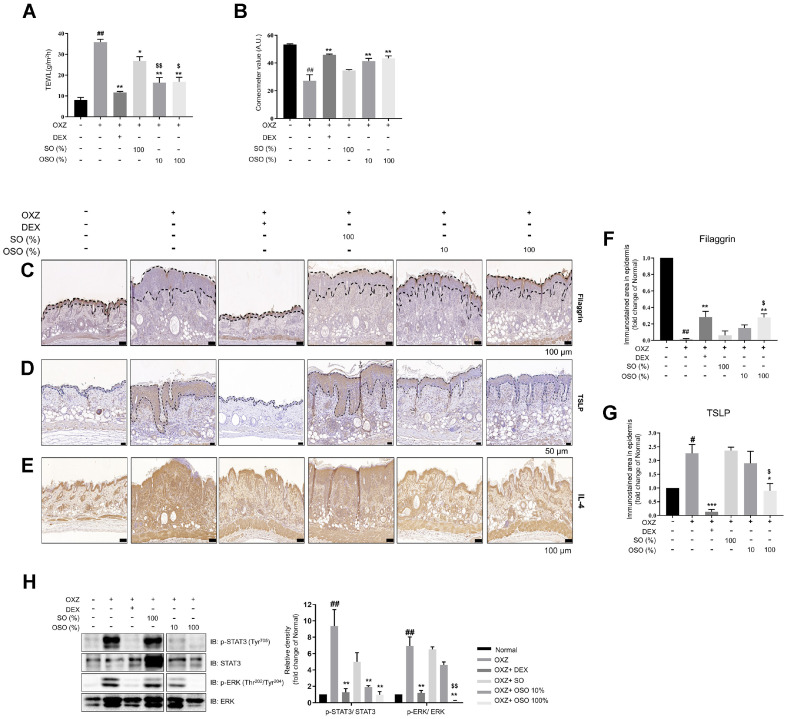
OSO treatment restores the skin barrier function in an AD-like hairless mouse model. A-B. At the end of the experiment, TEWL (**A**) and hydration (**B**) levels in the AD-like mouse skin lesions were measured. **C–G**. AD skin lesion filaggrin (Scale bar, 100 μm) (**C**), TSLP (Scale bar, 50 μm) (**D**), and IL-4 (Scale bar, 100 μm) (**E**) expression levels were analyzed using DAB staining. Analysis of epidermis filaggrin (**F**) and TSLP (**G**) DAB staining intensity was performed using Image (**J**). (**H**) Quantification of the protein ratios p-STAT3 (Tyr^705^)/STAT3 and p-ERK(Thr^202^/Tyr^204^)/ERK in AD-like mouse skin lesion. The results are expressed as the mean ± SEM (*n* = 3–4 per group). *, *p* < 0.05; **, *p* < 0.01 compared with the OXZ-only group. ^$^, *p* < 0.05; ^$$^, *p* < 0.01 compared with the OXZ + SO group. ^##^, *p* < 0.01 compared with the normal group (A, B, F, G, and H).

The transepidermal water loss (TEWL) in the OXZ-only group increased 4.45-fold compared with the normal group, while the TEWL in the OSO groups (10% and 100% OSO) showed a significant reduction compared with the OXZ-only group (Fig. 3A). In addition, the skin hydration level of the OSO groups (10% and 100% OSO) increased compared with the OXZ-only group (Fig. 3B). Filaggrin expression increased in the groups that received 10% and 100% OSO. On the other hand, TSLP and IL-4 levels decreased in the skin of the OXZ + 100% OSO group compared to the OXZ-only group (Fig. 3C–3G). The phosphorylation of STAT3 and ERK was significantly reduced in the skin of the OSO-administrated group compared to the OXZ-only group (Fig. 3H). Overall, these results suggest that OSO can contribute to the restoration of skin barrier through the IL-4-STAT3-ERK pathways.

### Antioxidant and Anti-Inflammatory Effects of OSO in the OXZ-Induced AD-Like Mouse Model

**Fig. 4:**
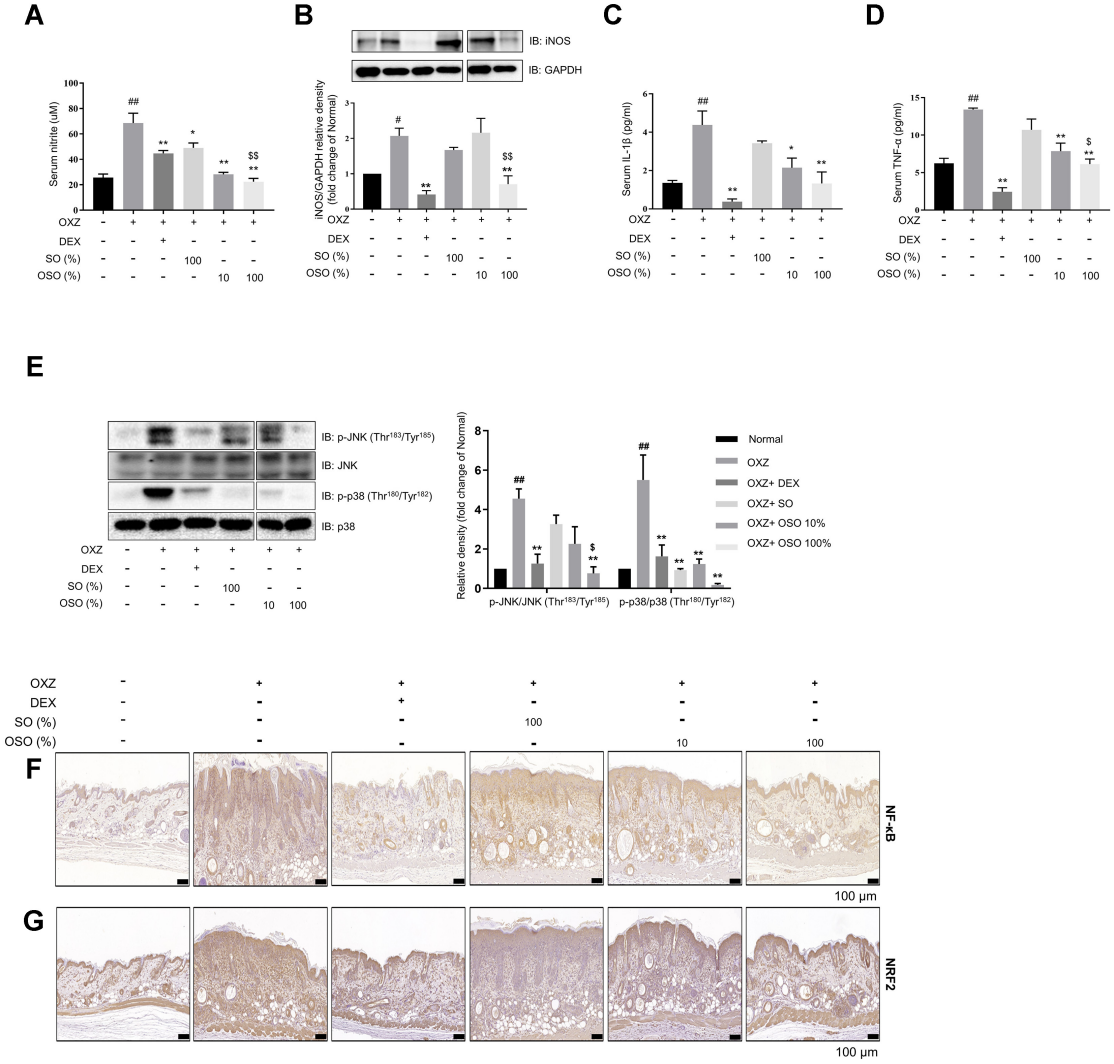
OSO exerts anti-inflammatory and antioxidant effects in AD-like hairless mice. (**A**) The effect of OSO on serum NO levels was determined using NO assay. (**B**) The iNOS/GAPDH ratio in skin tissue. (**C-D**) IL-1β (**C**) and TNF-α (**D**) levels in the serum were determined using ELISA. (**E**) Quantification of the protein ratios p-JNK (Thr^183^/Tyr^185^)/JNK and p-p38 (Thr^180^/Tyr^182^)/p38. (**F–G**) NF-κB (**F**) and NRF2 (**G**) on an AD skin lesion (Scale bar, 100 μm) were confirmed using DAB staining. The results are expressed as the mean ± SEM (*n* = 3–4/group). *, *p* < 0.05; **, *p* < 0.01 compared with the OXZ-only group. ^$^, *p* < 0.05; ^$$^, *p* < 0.01 compared with the OXZ + SO group. ^##^, *p* < 0.01 compared with the normal group (**A–E**).

It is well known that NO is a signaling molecule that plays a key role in the pathogenesis of inflammation [28]. As shown in Fig. 4A–4B, the levels of NO and inducible NO synthase (iNOS) expression decreased in the serum and skin of the OXZ + 100% OSO group, respectively, compared to the OXZ-only group. Additionally, the serum levels of IL-1b and TNF-α also decreased (Fig. 4C–4D). Furthermore, the phosphorylation of MAPK family kinases, including c-Jun N-terminal kinase (JNK) and p38, decreased in the skin of the OXZ + 100% OSO group compared to the OXZ-only group (Fig. 4E). NF-κB is known as a regulator of immune and inflammatory responses [29, 30]. The expression of transcription factor nuclear factor erythroid 2-related factor 2 (NRF2) is elevated in AD, and it is associated with skin barrier defects and inflammatory responses [31]. Histological analysis revealed that OSO treatment prevented the OXZ-mediated upregulation of NF-κB and NRF2 expression (Fig. 4F–4G). Taken together, these observations indicate that the improvement effect of OSO on AD is mediated by its antioxidant and anti-inflammatory activities.

## Discussion

In this study, we found that OSO alleviated the symptoms of AD in both OXZ-induced AD-like mouse model and LPS-treated RAW 264.7 cells. The inhibitory effects of OSO on AD was associated with its anti-inflammatory and antioxidant activities.

OSO alleviated the dermatitis score, hyperkeratosis, and epidermal thickness (Fig. 2B, 2C, 2E, and 2F). In addition, the number of infiltrating mast cells and IgE levels were reduced after the application of OSO (Fig. 2D, 2G, and 2H). These findings suggest that OSO has the potential to prevent AD-related barrier destruction by inhibiting IgE-mediated mast cell activation.

The disruption of the skin barrier triggers keratinocytes to release TSLP, which is a Th2-immune inducing factor [32]. TSLP causes pruritus and skin thickening in the pathogenesis of AD. Soumelis *et al*. reported that, the overexpression of TSLP in the keratinocytes of both acute and chronic lesions was observed in AD patients, but not in disease-free individuals [33]. Its expression was correlated with the Scoring AD Index, indicating that increased expression of TSLP corresponds to impaired skin barrier function [34]. Interestingly, as shown in Fig. 3D and 3G, TSLP expression decreased in the skin of the OXZ + 100% OSO group compared to the OXZ-only group. These results support the idea that OSO has a protective effect against OXZ-induced skin barrier damage.

The Janus kinase (JAK)-STAT pathway (JAK-STAT pathway) is one of the most important signal transduction pathways that mediate inflammatory signals [35]. Upon ligand binding, JAKs are activated and phosphorylate downstream STATs. The phosphorylated STATs are activated and translocate from the cytoplasm to the nucleus, where they act as transcription factors to regulate cytokine transcription [36]. In the epidermal keratinocytes of AD, IL-4 binds to its receptor, IL-4Rα, and activates STAT3 to promote the transcription of TSLP [37, 38]. TSLP inhibits the expression of filaggrin by up-regulating the STAT3/ERK pathway, leading to AD progression [16, 39]. Through further molecular analysis, we confirmed that OSO inhibited the downregulation of filaggrin following OXZ treatment (Fig. 3C and 3F) and the phosphorylation of STAT3 and ERK (Fig. 3H). This indicates that protective effects of OSO on the skin barrier are mediated through the inhibition of the IL-4/STAT3 signaling pathway.

Oxidative stress is defined as the formation of oxidants that exceed the antioxidant defense capacity in cells [40]. Antioxidants are chemicals that neutralize and prevent oxidative damage to vital cellular components, such as DNA, proteins, and the cell membrane [41, 42]. Free radicals are primarily associated with oxidative stress. As shown in Fig. 1A, we confirmed the potent free-radical scavenging capabilities of OSO using the DPPH assay, indicating that OSO has a strong capacity to alleviate oxidative stress. Also, oxidative stress significantly promotes inflammation in the skin by upregulating pro-inflammatory cytokines [43]. The level of NO and iNOS, trigger of oxidative stress, was elevated in our AD-like models. Interestingly, OSO treatment reversed excessive NO production (Fig. 1D and 4A-4B). These results suggest that OSO has antioxidant effects.

O3 exerts anti-inflammatory effects by inhibiting NF-κB and reducing proinflammatory cytokines such as TNF-α and IL-1β [44]. It is also well known that the MAPK family, including p38 and JNK, plays an important role in regulating IL-1β and TNF-α-mediated inflammation [45]. In this study, levels of IL-1β and TNF-α decreased after administration of OSO (Fig. 1E and 4C–4D). At the same time, phosphorylation of p38 and JNK, as well as NF-κB expression, were down-regulated (Fig. 4E–4F). These results suggest that OSO suppressed the production of IL-1β and TNF-α by inhibiting the p38 and JNK signaling pathway.

The epidermis maintains internal redox-sensitive pathways that adeptly respond to detrimental stimuli from the surrounding environment. The role of NRF2 is pivotal in creating and upholding the thiol gradient, providing indispensable cellular protection [46]. However, according to research by Ogaw T *et al*. [31], NRF2 is upregulated due to the inflammatory response caused by skin hapten penetration and the destruction of the skin barrier. Indeed, they confirmed that NRF2 is upregulated in AD patients. OSO appears to contribute to reducing inflammatory responses in AD by decreasing this overexpression of NRF2 through the restoration of the skin barrier (Fig. 4G).

Other factors that contribute to the onset of AD include dysbiosis of the skin microbiota (particularly overgrowth of *S. aureus*) [47]. O_3_ therapy can deactivate various pathogens including bacteria, fungi, and viruses [44]. In a previous study, we found that OSO inhibits the growth of *S. aureus* [23]. These findings support our hypothesis that the topical application of OSO could improve AD. Therefore, we propose that OSO is an exceptional adjuvant with anti-inflammatory and antioxidant properties that can restore the skin’s barrier function and improve symptoms of AD.

## Conclusion

Our results indicated that OSO could suppress inflammation in an OXZ-induced AD mouse model and LPS-treated RAW 264.7 cells by decreasing pro-inflammatory, Th2 cytokine responses and promoting antioxidant activity. Moreover, OSO protected against OXZ-induced skin barrier damage by increasing filaggrin expression through the inhibition of the IL-4/STAT3/MAPK pathway and by decreasing TSLP expression. These findings suggest that OSO may be a potential treatment for AD.

## Figures and Tables

**Table 1:** Primary antibodies used in this study.

Antibodies	Product code	Company
Anti-Filaggrin	GTX37695	Gene Tex
Anti-TSLP	ab188766	Abcam
Anti-IL-4	MBS2027100	My Bio Source
Anti-NF-kB	8242S	Cell Signaling Technology (CST)
Anti-GAPDH	5174S	CST
Anti-phosphor(p)-ERK	9101S	CST
Anti-ERK	9102S	CST
Anti-p-JNK	9251S	CST
Anti-JNK	SC-7345	Santa Cruz Biotechnology
Anti-p-p38	4511S	CST
Anti-p38	9212S	CST
Anti-p-STAT3	9131S	CST
Anti-STAT3	9139S	CST
Anti-iNOS	610328	BD Biosciences
Anti-NRF2	PA5-27882	Invitrogen
